# The Role of Macrophages in the Pathogenesis of ALI/ARDS

**DOI:** 10.1155/2018/1264913

**Published:** 2018-05-13

**Authors:** Xiaofang Huang, Huiqing Xiu, Shufang Zhang, Gensheng Zhang

**Affiliations:** ^1^Department of Critical Care Medicine, Second Affiliated Hospital, Zhejiang University School of Medicine, Hangzhou, Zhejiang 310009, China; ^2^Department of Cardiology, Second Affiliated Hospital, Zhejiang University School of Medicine, Hangzhou, Zhejiang 310009, China

## Abstract

Despite development in the understanding of the pathogenesis of acute lung injury (ALI)/acute respiratory distress syndrome (ARDS), the underlying mechanism still needs to be elucidated. Apart from leukocytes and endothelial cells, macrophages are also essential for the process of the inflammatory response in ALI/ARDS. Notably, macrophages play a dual role of proinflammation and anti-inflammation based on the microenvironment in different pathological stages. In the acute phase of ALI/ARDS, resident alveolar macrophages, typically expressing the alternatively activated phenotype (M2), shift into the classically activated phenotype (M1) and release various potent proinflammatory mediators. In the later phase, the M1 phenotype of activated resident and recruited macrophages shifts back to the M2 phenotype for eliminating apoptotic cells and participating in fibrosis. In this review, we summarize the main subsets of macrophages and the associated signaling pathways in three different pathological phases of ALI/ARDS. According to the current literature, regulating the function of macrophages and monocytes might be a promising therapeutic strategy against ALI/ARDS.

## 1. Introduction

Acute lung injury (ALI)/acute respiratory distress syndrome (ARDS) is a devastating respiratory disorder, which leads to mortality in patients in intensive care units [1]. It is characterized by clinically significant hypoxemia, diffuse bilateral pulmonary infiltration, pulmonary edema, a decrease in pulmonary compliance, and a decrease in the functional residual capacity [2]. Pathological changes include increased vascular permeability caused by alveolar-capillary membrane dysfunction, with flooding of protein-rich fluid, alveolar hemorrhage, and fibrin deposition [3]. ALI/ARDS develops by excessive and uncontrolled systemic inflammatory responses to direct or indirect lung injury. Currently, there is increasing evidence suggesting that macrophages, including resident alveolar macrophages (AMs) and recruited macrophages from the blood, are key factors in the pathogenesis of ALI/ARDS [4, 5]. The role of macrophages during development of the inflammatory response is subtle. In general, they exert a proinflammatory effect in the early stage and exhibit an anti-inflammatory effect in the late stage. These effects may be attributed to the phenotypic transformation, which is in part regulated by the suppressor of cytokine signaling (SOCS) 1/SOCS3 and interferon regulatory factor (IRF) 4/IRF5 [6–9]. In this review, we summarize the main subsets of macrophages involved in ALI/ARDS and the recent advances in the phenotypic and functional alterations. The identification of the cellular and molecular mechanisms associated with the role of macrophages in ALI/ARDS will provide a basis for some potential treatment strategies.

## 2. Main Macrophages Involved in ALI/ARDS

Classified by the responses to environmental stimuli, there are two polarization states of macrophages: the classically activated phenotype (M1) and the alternatively activated phenotype (M2) [10].

There are two main types of macrophages in the alveolus. The first type is the long-lived resident AMs, which are located at the air-tissue interface, with an approximate density of seven per alveolus [11]. As a predominant cell type in the alveolar airspaces, they act as a uniform, quiescent, and immunosuppressive population in the normal state [12, 13]. The M2 phenotype is the main form of these resident AMs. The second type is the recruited AMs. When a stimulus occurs, such as in ALI/ARDS, peripheral blood monocytes are recruited into the alveolar lumen, where they differentiate into macrophages with the M1 phenotype [11, 14].

To further investigate the role of AMs in the pathogenesis of ALI/ARDS, clodronate-loaded liposomes have been used to eliminate AMs specifically in blockage experiments [15, 16]. Other studies have shown that the depletion of AMs significantly reduced pulmonary edema and ventilator-induced lung injury in rats [17, 18], which was partially attributed to the decreased recruitment of neutrophils in the lungs. Besides, depletion of AMs also has been found [19] to mitigate lung injury significantly at 4 h after lipopolysaccharide (LPS) administration in mice by attenuating neutrophilic alveolitis and reducing proinflammatory cytokines.

Machado-Aranda et al. [20] have shown that neutrophils were significantly recruited to the bronchoalveolar lavage fluid from 5 h after contusion-induced lung injury in the AM-depleted group, which resulted in a worsened pulmonary compliance, an increased lung permeability, and aggressively elevated proinflammatory cytokine levels. In a study by Broug-Holub et al. [21], substantial neutrophil recruitment and decreased bacterial clearance were found in the lungs of AM-depleted mice at 48 h after infection with *Klebsiella pneumoniae*. In addition, Narasaraju et al. [22] have shown that influenza infection led to excessive recruitment of neutrophils, extensive alveolar damage, and increased viral load in the AM-depleted group at 5 days after infection.

**Figure 1:**
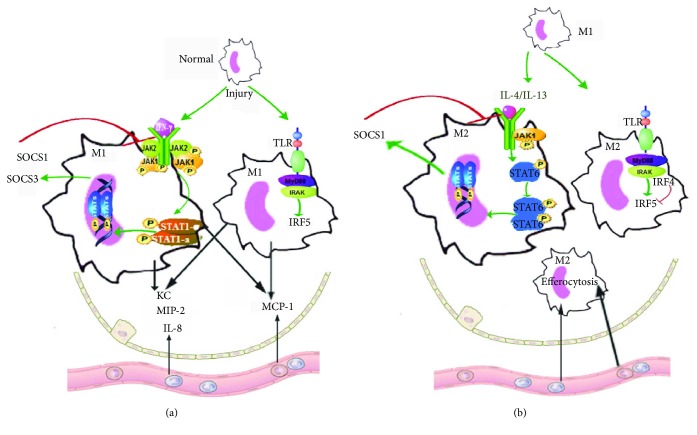
The molecular mechanism of SOCS- and IRF-regulated cytokine signaling in macrophages during ALI/ARDS. (a) Normal resident AMs are activated and shift into the M1 phenotype upon certain stimulation during the exudative phase of ALI/ARDS. Proinflammatory cytokines such as IFN-*γ*, TNF-*α*, and IL-*β* are excreted by M1 macrophages into the site of inflammation. The JAK–STAT1 pathway is activated by IFN-*γ*, and SOCS1 and SOCS3 are induced. SOCS1 and SOCS3 inhibit the signaling pathway by different mechanisms. IRF5 promotes M1 polarization by directly binding to IL-12 and IL-23 promoters. Leukocytes migrate into the cellular airspace by the activation of chemokines such as KC, MIP-2, and IL-8. Monocytes from the circulation are also recruited by chemokines such as MCP-1 and shift into the M1 phenotype. Crosstalk between SOCS3 and IRF5 may exist. (b) Macrophages shift from the M1 phenotype to the M2 phenotype during the later phase of ALI/ARDS. This process is regulated by several factors, including IL-4, IL-10, IL-13, STAT6, and IRF4. IL-4 or IL-13 activates the JAK–STAT6 pathway, and SOCS1 is induced. SOCS1 feedback inhibits the IL-4/IL-13 signaling. IRF4 inhibits IRF5 activation by a competing interaction with the adaptor MyD88. Recruited macrophages play an important role in eliminating apoptotic cells, debris, and pathogens. AMs: alveolar macrophages; IFN-*γ*: interferon-*γ*; JAK: Janus kinase; IL: interleukin; STAT: signal transducer and activator of transcription; IRF: interferon regulator factor; SOCS: suppressors of cytokine signaling; KC: keratinocyte-derived chemokine; MIP: macrophage inflammatory protein; MCP: monocyte chemoattractant protein; TNF-*α*: tumor necrosis factor *α*; TLR: toll-like receptor; MyD88: myeloid differentiation factor 88.

According to these results from various experimental models, AM depletion shows a protective effect against ALI/ARDS at an early stage, while it exacerbates lung injury at a later stage. Neutrophils are the first leukocytes recruited to the sites of inflammation in response to chemokines such as macrophage inflammatory protein-2 (MIP-2) and interleukin- (IL-) 8 released by activated AMs [23–26]. The accumulation of neutrophils is an important factor leading to tissue damage due to the overwhelming release of cytotoxic and proinflammatory mediators [27]. At an early stage, AM depletion may alleviate neutrophil-induced alveolitis by reducing inflammatory responses. At a later stage, circulating monocytes migrate into the interstitium or alveolar spaces in response to monocyte chemoattractant protein-1 (MCP-1) for pathogen clearance and phagocytosis of infected particles or other inflammatory debris (Figure 1(a)) [13, 28]. Macrophage phagocytosis of neutrophils and other apoptotic cells is an important step in the process of inflammatory response regulation. At this stage, the number of recruited AMs is increased, and the depletion of AMs may result in decreased phagocytic macrophages and control of inflammation. On the other hand, intraperitoneal administration of MCP-1 significantly increased the total number of macrophages as well as the number of phagocytizing neutrophils [29]. The increased chemokine ligand 2 (CCL-2 or MCP-1) expression in turn enhances CC chemokine receptor 2 (CCR2) expression in circulating monocytes and interstitial macrophages [30]. The interaction of CCL-2 with CCR2 plays an important role in the process of reepithelialization after lung injury [31]. In mice subjected to intratracheal LPS treatment, CCR2 deficiency aggravated the apoptosis of alveolar epithelial cells and permeability injury [32]. Therefore, these two types of AMs act at an early or a late stage in the process of ALI/ARDS: the resident AMs release cytokines for recruiting neutrophils or monocytes to promote and sustain inflammation, and the recruited AMs mainly function to clear pathogens and limit inflammatory responses.

Similarly, the role of circulating monocytes has been investigated in LPS-induced lung injury models. Herold et al. [32] have reported that depletion of circulating monocytes (the precursors of exudate macrophages) seems to aggravate LPS-induced lung injury. In contrast, Dhaliwal et al. [33] have found that depletion of the pool of peripheral blood monocytes might alleviate lung injury and might be a therapeutic target for ALI/ARDS. These controversial results may be due to the different times of monocyte depletion. In the study by Herold et al., depletion of circulating monocytes was performed at 12 h before LPS challenge [32]. Meanwhile, in the study by Dhaliwal et al., the process was initiated by the use of clodronate at 6 h after intratracheal LPS administration [33]. Taken together, AMs and peripheral blood monocytes exert distinctly exclusive functions at different stages of ALI/ARDS [34, 35]. The modulation of macrophages and monocytes may be a potential way to treat ALI/ARDS. However, the mechanism of cellular interactions between macrophages and several anti-inflammatory cells remains unclear [36].

## 3. Phenotype and Functional Alternation of Macrophages in the Pathogenesis of ALI/ARDS

### 3.1. Exudative Phase of ALI/ARDS

In healthy lungs, resident AMs are highly plastic for immune responses, with relatively high expression levels of pathogen-associated molecular pattern and danger-associated molecular pattern receptors [37]. Upon stimulation in ALI/ARDS, resident AMs immediately shift to the predominant M1 phenotype in response to infection-induced activation of toll-like receptors (TLRs) or other recognition receptors [38]. These AMs act as the first line of defense against pathogenic microorganisms and lung injury, such as bacteria, endotoxins, viruses, and ventilator-induced lung injury [22, 39, 40], releasing various potent proinflammatory and deleterious mediators including IL-1*β*, IL-6, and IL-18 [41, 42]. With these inflammatory factors, the neutrophils are recruited from the intravascular space, across the endothelium and epithelium, and finally into the lungs and alveolar space. It is widely known that excessive accumulation of proinflammatory cytokines and neutrophils is involved in the pathophysiology of tissue damage in inflammatory diseases [43]. Therefore, M1 macrophages serve as a promoter in the process of lung tissue damage in ALI/ARDS. However, a protective effect associated with M1 macrophages also has been detected. Recent studies have found that these macrophages generate a high level of amphiregulin [44, 45], a ligand of epithelial growth factor receptor, which has been found to protect the epithelial barrier and inhibit the gene expression of proinflammatory cytokines in LPS-induced ALI and ventilator-induced lung injury models [44, 45].

The shift of macrophage phenotypes is regulated by several signaling pathways (Figure 1(a)). M1 macrophages can be activated through the classical JAK/STAT1 pathway [46–48]. In detail, interferon-*γ* (IFN-*γ*) binds to the receptors on the cell surface, activating Janus kinase 1 (JAK1), JAK2, and signal transducer and activator of transcription 1 (STAT1) [46]. In addition, STAT expression is negatively regulated, in part, via SOCS [49]. Under normal conditions, the expression levels of SOCS1 and SOCS3 are very low, but they are rapidly activated by IFN-*γ* or LPS [6]. Specifically, SOCS1 and SOCS3 negatively regulate the JAK/STAT pathway through binding with the key phosphorylated tyrosine residues of JAKs and/or cytokine receptors [50, 51]. Besides, other possible mechanisms accounting for SOCS proteins inhibiting JAK/STAT signaling also have been identified [52, 53]. In mouse macrophage cells, the downregulation of SOCS1 expression by SOCS1 short hairpin (sh) RNA transfection significantly increased the mRNA levels of JAK1 and STAT1 as well as promoted the polarization of macrophages to the M1 phenotype [54]. In addition, Qin et al. [55] have revealed that myeloid-specific SOCS3-deficient mice exhibit enhanced activities of STAT1/3 and increased plasma levels of proinflammatory cytokines and chemokines. Therefore, both SOCS1 and SOCS3 inhibit the polarization of macrophages into the M1 phenotype, decreasing proinflammatory chemoattractants. Additionally, M1 macrophages have shown an impaired proinflammatory effect and an enhanced anti-inflammatory effect in the absence of SOCS3 in a nephrotoxic nephritis model [7], thus indicating an essential role of SOCS3 in the development of M1 macrophages. However, whether similar mechanisms exist in ALI/ARDS models remains to be further investigated.

The transcription factor IRF5 is another major regulator of proinflammatory M1 macrophage polarization. It is generally involved in the process of the downstream TLR-myeloid differentiation factor 88 signaling pathway, inducing proinflammatory cytokines and repressing transcription of anti-inflammatory cytokines such as IL-10 [56]. In a study by Qin et al. [57], IRF5 expression was increased in the absence of SOCS3, which promoted M1 macrophage polarization. Thomas et al. [8] also have shown that IRF5 deficiency led to a lower proportion of M1 macrophage subsets in mice and a lower expression of M1-specific cytokines in human M1 macrophages [8]. Overall, STAT1, SOCS1/SOCS3, and IRF5 are involved in the polarization of M1 macrophages, with substantial crosstalk among these signaling pathways [57–60].

### 3.2. Rehabilitation Phase of ALI/ARDS

After pathogenic factors are eliminated, resident and recruited macrophages shift from the M1 phenotype to the anti-inflammatory M2 phenotype [5]. According to various activating conditions, M2 macrophages are classified into four subtypes: M2a (IL-4- or IL-13-induced), M2b (immune complexes in combination with IL-1*β* or LPS), M2c (IL-10, transforming growth factor (TGF)-*β*, or glucocorticoids), and M2d (adenosine A_2A_ receptor agonists) [61–63]. M2 macrophages play an important role in lung tissue repair by limiting the levels of proinflammatory cytokines in the cellular space. They also help produce anti-inflammatory cytokines such as IL-10 and IL-1 receptor antagonist in response to T-helper 2 (Th2) cytokines [5, 35]. Apart from the balance of pro- and anti-inflammatory cytokines, the clearance of neutrophils from inflammatory sites is another contributing factor for rehabilitation [64]. Once recruited, neutrophils exhibit apoptosis and the apoptotic cells accumulate [65]. With a potent phagocytic capacity, M2 macrophages remove the necrotic cells and debris. The elimination of apoptotic cells by phagocytes, known as efferocytosis, can activate anti-inflammatory signaling and terminate proinflammatory responses [66]. Phagocytosis of apoptotic neutrophils by M2 macrophages further increases the levels of IL-10 and TGF-*β*1 [35, 67], which may help to control inflammation. It also inhibits the expression of inducible nitric oxide synthase and stimulates the expression of arginase 1, thereby preventing reactive nitric oxide production [5]. Besides, M2-derived cytokines, including IL-4, IL-13, and IL-10, further enhance efferocytosis, with an increased level of mannose receptor expression [68, 69]. With this positive feedback loop, efferocytosis and M2 polarization alleviate inflammation. However, it is still unclear how M2 differentiation is terminated and which mediators are involved.

In addition, recent studies have indicated that regulatory T cells (Tregs), a subset of CD4^+^ lymphocytes, are involved in the phenotypic transformation of macrophages [70, 71]. Tregs may reduce lung inflammation by diminishing the elevation of macrophage proinflammatory cytokine levels and enhancing the efferocytosis of apoptotic neutrophils [72–74]. Taams et al. [74] have shown that human Tregs inhibit LPS-induced M1 monocyte proinflammatory responses and promote the M2 phenotype. Eliminating Tregs has resulted in sustained proinflammatory responses induced by LPS and reduced neutrophil apoptosis [73, 74]. Future studies investigating the possible mechanisms for Treg-mediated neutrophil apoptosis are on the way [75].

Several signaling pathways are involved in the phenotypic shift from M1 to M2 macrophages (Figure 1(b)). The alternative macrophages are activated by Th2-type cytokines, including IL-4 and IL-13 [76]. However, the main sources of IL-4 and IL-13 in the body are largely unknown. Produced by various innate cells, IL-4 and IL-13 have many similar effects on macrophages due to an identical receptor chain, IL-4R*α*. Of note, IL-4 can activate the insulin receptor substrate-2 signaling pathway through the *γ*c chain, while IL-13 cannot [77]. Besides, IL-4 and/or IL-13 activate STAT6 and increase the expression of STAT6-responsive genes such as arginase 1 and 15-lipoxygenase [6]. In addition to the regulatory effects on the activation of macrophages by the classical IFN-*γ*-inducible pathway, SOCS1 regulates the alternative IL-4-inducible pathway [78]. IL-4 induces SOCS1 expression in macrophages via STAT6 signaling, and SOCS1 feedback inhibits IL-4 signaling to limit the expression of STAT6-responsive genes and the alternative macrophage activation pathway [6, 78]. In a study by Liu et al. [7], SOCS3 suppression enhanced STAT3 activity; decreased the expression of IL-6, nitric oxide, and CD86; and increased the expression of mannose receptor and arginase, which promote the polarization of macrophages into the M2 phenotype. Their results suggest that the expression of SOCS3 maintains the activated phenotype M1 cells with proinflammatory properties and inhibits the phenotype with anti-inflammatory effects [7]. Taken together, SOCS1 and SOCS3 are involved in the classical macrophage activation pathways as well as in various alternative macrophage activation pathways. Further investigations to mitigate ALI/ARDS by regulating the balance between anti-inflammatory and proinflammatory effects of macrophages are needed.

IRF4, another member of the IRF family, has diverse effects in different situations. It inhibits IRF5 activation by a competing interaction with the adaptor myeloid differentiation factor 88 in resident peritoneal macrophages. However, in bone marrow-derived macrophages, this type of competition is greatly minimized due to the lack of IRF5-dependent TLR signaling [79]. Another study has suggested that IRF4 controls M2 macrophage polarization by stimulating the expression of specific M2 macrophage markers in mice [9]. From the current evidence, STAT6, SOCS1/SOCS3, and IRF4 are all involved in the polarization of M2 macrophages [58, 59].

### 3.3. Fibrotic Phase of ALI/ARDS

Pulmonary fibrosis, a late complication of ALI/ARDS, is marked by fibroblast proliferation and excessive deposition of extracellular matrix [80]. M2 phenotype cells are involved in regulating the fibrotic responses in the lungs [81, 82]. Persistence of M2 macrophages at the injury sites is a hallmark of the development of fibrosis, and the steady expression of IL-4 and IL-13 can promote collagen deposition through TGF-*β* and arginase 1 pathways [83, 84]. Wakayama et al. [85] have demonstrated that dental pulp stem cells can ameliorate bleomycin-induced lung injury and fibrosis by inducing anti-inflammatory M2-like lung macrophages. Moreover, recent studies have reported that IL-4-polarized M2 macrophages have the potential to limit fibrosis through expressing arginase 1 and resistin-like *α* (surface markers of the M2 phenotype) genes [78, 86]. In summary, macrophages are paradoxically involved in both the generation of pulmonary fibrosis and the later healing process [86]. They regulate fibroblast recruitment, growth, and connective-tissue remodeling; in addition, they contribute to the removal of dead tissue, the growth of new blood vessels, and fibrin dissolution [61]. The regulatory mechanism for the balance of positive and negative regulators on the profibrotic functions of macrophages needs to be clarified in the future.

## 4. Conclusion

In this review, we summarize the current research on the role of macrophages/monocytes in inflammation, tissue repair, and fibrosis in ALI/ARDS. In general, macrophages/monocytes exert a proinflammatory or an anti-inflammatory effect based on the microenvironment in different stages. Limiting excessive proinflammatory responses in the exudative phase and excessive fibroblast proliferation in the repair phase through the regulation of macrophage activation and polarization may be a novel therapeutic target for ALI/ARDS. However, there is still a long way to go.
